# Deletion of plasma *Phospholipid Transfer Protein (PLTP)* increases microglial phagocytosis and reduces cerebral amyloid-β deposition in the J20 mouse model of Alzheimer's disease

**DOI:** 10.18632/oncotarget.24802

**Published:** 2018-04-13

**Authors:** Marine Mansuy, Stella Baille, Geoffrey Canet, Amélie Borie, Catherine Cohen-Solal, Michel Vignes, Véronique Perrier, Nathalie Chevallier, Naig Le Guern, Valérie Deckert, Laurent Lagrost, Laurent Givalois, Catherine Desrumaux

**Affiliations:** ^1^ Université Montpellier, Montpellier, F-34095 France; ^2^ Team ‘Environmental Impacts in Alzheimer's disease and related disorders’ (EiAlz), Inserm, U1198, Montpellier, F-34095 France; ^3^ EPHE, Paris, F-75007 France; ^4^ LipSTIC LabEx, Fondation de Coopération Scientifique Bourgogne-Franche Comté, F-21000 Dijon, France; ^5^ UMR 5247, Max Mousseron Biomolecules Institute, 34095 Montpellier Cedex 5, France; ^6^ INSERM, LNC UMR1231, F-21000 Dijon, France; ^7^ University Bourgogne Franche-Comté, LNC UMR1231, F-21000 Dijon, France

**Keywords:** phospholipid transfer protein, Alzheimer’s disease, innate immunity, phagocytosis, microglia

## Abstract

Plasma phospholipid transfer protein (PLTP) binds and transfers a number of amphipathic compounds, including phospholipids, cholesterol, diacylglycerides, tocopherols and lipopolysaccharides. PLTP functions are relevant for many pathophysiological alterations involved in neurodegenerative disorders (especially lipid metabolism, redox status, and immune reactions), and a significant increase in brain PLTP levels was observed in patients with Alzheimer's disease (AD) compared to controls. To date, it has not been reported whether PLTP can modulate the formation of amyloid plaques, i.e. one of the major histopathological hallmarks of AD.

We thus assessed the role of PLTP in the AD context by breeding PLTP-deficient mice with an established model of AD, the J20 mice. A phenotypic characterization of the amyloid pathology was conducted in J20 mice expressing or not PLTP. We showed that PLTP deletion is associated with a significant reduction of cerebral Aβ deposits and astrogliosis, which can be explained at least in part by a rise of Aβ clearance through an increase in the microglial phagocytic activity and the expression of the Aβ-degrading enzyme neprilysin.

PLTP arises as a negative determinant of plaque clearance and over the lifespan, elevated PLTP activity could lead to a higher Aβ load in the brain.

## INTRODUCTION

Plasma phospholipid transfer protein (PLTP) belongs to the lipid transfer/lipopolysaccharide binding protein (LT/LBP) gene family as do cholesteryl ester transfer protein (CETP) and two proteins involved in innate immunity, lipopolysaccharide binding protein (LBP) and bactericidal permeability increasing protein (BPI) [1]. PLTP is ubiquitously expressed in vertebrate species, and has the ability to bind and transfer a number of amphipathic compounds, including phospholipids, unesterified cholesterol, diacylglycerides, tocopherols and lipopolysaccharides (LPS) [2]. Many studies have been conducted to decipher the complex action of PLTP on lipid and lipoprotein metabolism. The relationship of PLTP to atherosclerosis is rather puzzling and may be dependent on the experimental setting, the animal model used and the PLTP source (plasma versus macrophage-derived) [3, 4]. Through its ability to transport the lipophilic antioxidant alpha-tocopherol, *i.e.* the main isomer of vitamin E, PLTP also plays a major role in maintaining the redox equilibrium between the intravascular compartment and tissues, with reported pathophysiological implications in the fields of vascular homeostasis, fertility, and cognitive functions [5–9]. Last but not the least, PLTP has recently been reported to modulate inflammation and immune responses. These actions rely both on its ability to transport and detoxify lipopolysaccharides (the main component of the cellular wall of Gram-negative bacteria) [10–12] and on its direct action on immune cell functions [13, 14]. Recently, a positive relationship was established between PLTP activity and inflammatory markers in patients with cardiovascular disease or diabetes [15–17].

PLTP is secreted by a wide variety of tissues. It is highly expressed in the central nervous system (CNS), and its activity can be measured in cerebrospinal fluid (CSF) [18, 19]. PLTP functions are relevant for many pathophysiological alterations involved in the development and progression of neurodegenerative disorders (especially lipid metabolism, redox status, and immune reactions [20–22]), and a significant increase in brain PLTP levels, along with a decrease of its activity in the CSF were observed in patients with Alzheimer's disease (AD) compared to controls [19, 23].

AD is the leading cause of dementia in the elderly population, and is characterized by the accumulation and deposition of toxic amyloid-beta peptides (Aβ) in the brain tissue along with a neuroinflammatory reaction. Although a previous work from our group demonstrated that PLTP deficiency in mice exacerbates Aβ neurotoxicity and associated cognitive loss through a significant increase in cerebral oxidative stress [7], it has not been reported whether PLTP can modulate the formation of amyloid plaques, *i.e.* one of the major histopathological hallmarks of AD.

To address this question and clarify the functional importance of PLTP in the pathophysiology of AD, we have undertaken in the present work an *in vivo* assessment of the role of PLTP in the AD context by breeding PLTP-deficient mice with an established model of AD, the J20 mice. These mice overexpress the hAPP gene bearing the Swedish and Indiana mutations, under the control of the PDGF promoter [24]. We demonstrate that PLTP deficiency in J20 mice strongly reduces amyloid-beta peptide deposition through an upregulating effect on Aβ clearance mechanisms involving microglial phagocytosis and neprilysin activity. Although amyloid plaques were reduced, soluble amyloid-beta peptide amounts were comparable and spatial memory was not significantly improved in PLTP-deficient compared to wild-type mice in the J20 line, which argues against a direct relationship between amyloid deposition and cognitive dysfunction in this model.

## RESULTS

### PLTP deletion does not alter soluble Aβ levels in TgAPP mice

To address the impact of PLTP on pathways involved in AD, we generated TgAPP mice lacking PLTP (TgAPP/PLTP–/–) and compared them with their TgAPP littermates at the age of 6 months.

**Figure 1:**
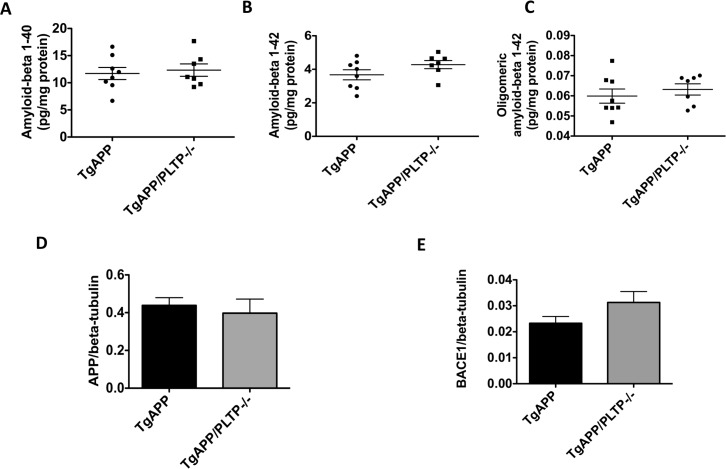
Human APP expression and processing in transgenic mice with or without PLTP deficiency (**A**–**C**) Soluble forms of amyloid-beta 1–40, amyloid-beta 1–42, and oligomeric amyloid-beta 1–42 were assayed in hemibrain homogenates from 6 months old TgAPP mice (*n* = 8) and TgAPP/PLTP–/– mice (*n* = 7) using ELISA kits, and results were normalized to protein concentrations. (**D**–**E**) Expression of the human APP transgene and of the APP-cleaving enzyme BACE1 was determined in hemibrain homogenates from TgAPP mice (*n* = 8) and TgAPP/PLTP–/– mice (*n* = 7) by Western Blot and normalized to the expression of the loading control beta-tubulin. Results are presented as mean ± SEM.

We first measured Aβ_1–40_ and Aβ_1–42_ levels in the soluble fraction of hemibrain homogenates. As shown in Figure 1A and 1B), both peptides were found in similar amounts in brains of TgAPP/PLTP–/– mice and their TgAPP littermates. A tendency towards an increase in Aβ_1–42_ levels was observed in TgAPP/PLTP–/– mice (*p* = 0.15). Oligomeric forms of the Aβ peptides, measured using the specific A11 antibody, were present at similar levels in the soluble fraction of brain homogenates from TgAPP and TgAPP/PLTP–/– animals (Figure 1C).

As shown in Figure 1D and 1E), the expression of the human APP transgene was not altered by PLTP deletion. A non significant increase (*p* = 0.11) in the amount of the APP-cleaving enzyme BACE1 was measured in brain homogenates of TgAPP/PLTP–/– compared to TgAPP mice, suggesting the amyloidogenic processing of APP is not significantly altered by PLTP deletion.

### PLTP deletion decreases Congo-Red positive plaque load in TgAPP mice

**Figure 2:**
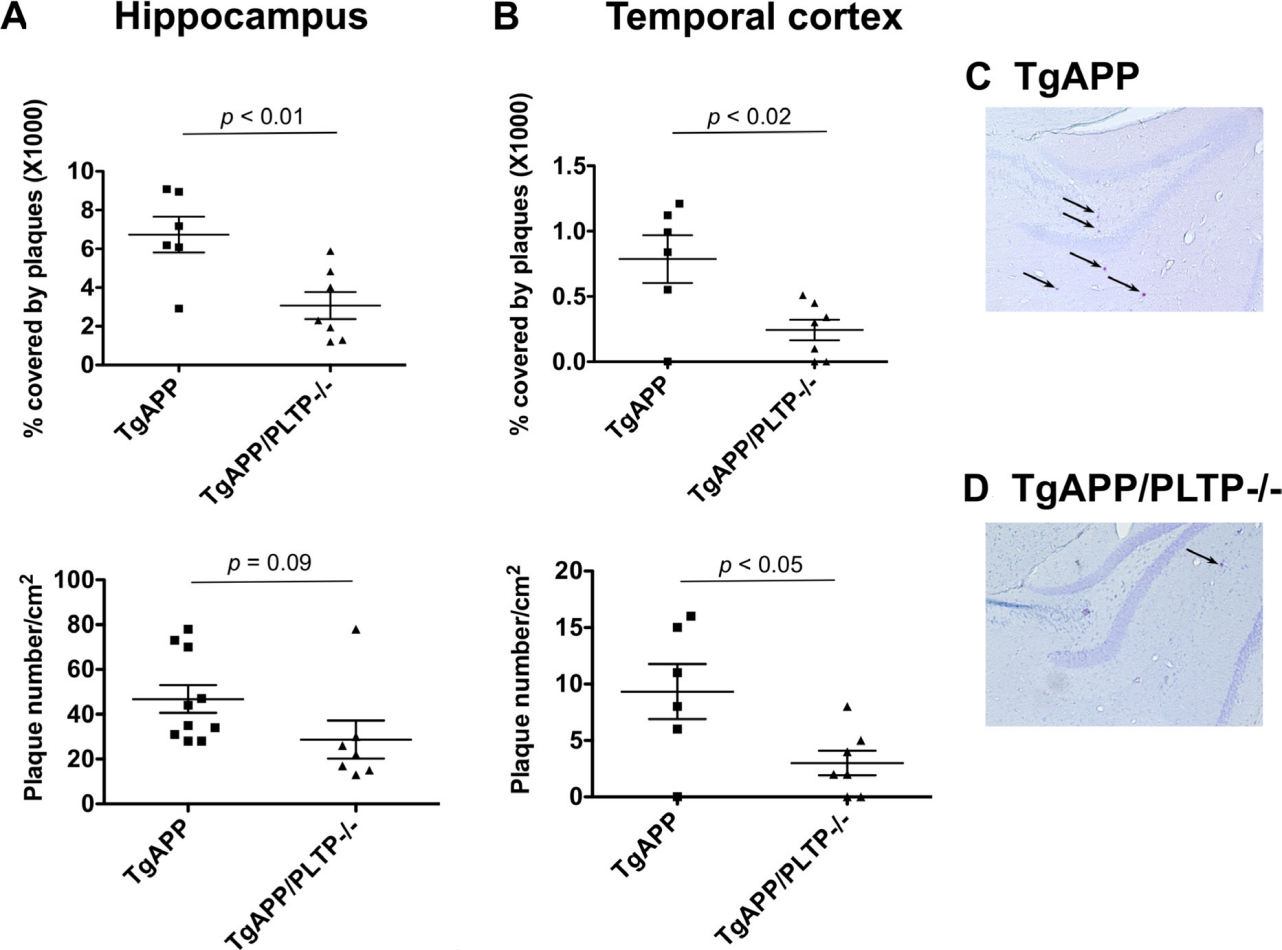
Quantification of Congo-Red positive plaques in TgAPP and TgAPP/PLTP–/– mice Brain sections from 6 months old TgAPP mice (*n* = 10) and TgAPP/PLTP–/– mice (*n* = 7) were stained with Congo Red and images were captured using an optical microscope. Panels (**A–D**) Amyloid plaques counts and surfaces were determined and normalized to the total surface of the hippocampus or temporal cortex. Results are presented as mean+SEM. Statistical differences were assessed using the Student’s *t* test. Panels (**C**–**D**) Representative pictures showing Congo-Red positive plaques (black arrows) in the hippocampus of TgAPP and TgAPP/PLTP–/– mice. Scale bar, 500 μm.

To address the impact of PLTP on amyloid-beta peptide deposition, the number and size of Congo-Red positive amyloid plaques were measured in the hippocamus and in the temporal cortex of TgAPP and TgAPP/PLTP–/– mice at the age of 6 months. As shown in Figure 2A, 2B, upper panels), hippocampal and cortical Congo Red-positive plaque surfaces, expressed as a percentage of the analyzed area, were markedly decreased in PLTP-deficient compared to PLTP-expressing animals (x1000 values, hippocampus : 6.73 ± 0.93% in TgAPP (*n* = 10) *vs* 3.07 ± 0.70% in TgAPP/PLTP–/– mice (*n* = 7), *p* < 0.01; temporal cortex : 0.78 ± 0.18% in TgAPP (*n* = 10) *vs* 0.24 ± 0.08% in TgAPP/PLTP–/– mice (*n* = 7), *p <* 0.02). The number of amyloid plaques (lower panel) was significantly reduced in the temporal cortex of TgAPP/PLTP–/– mice (*n* = 6) compared to TgAPP mice (*n* = 7) (9.5 ± 2.4/cm^2^ *vs* 2.9 ± 1.1/cm^2^, respectively, *p* < 0.05), and a non significant decrease was measured in the hippocampus (46.9 ± 6.2/cm^2^ in TgAPP (*n* = 10) *vs* 28.8 ± 8.5/cm^2^ in TgAPP/PLTP–/– mice (*n* = 7), *p* = 0.09) (Figure 2A, 2B, lower panels). It is worthy of note that as previously reported in this model, amyloid plaques were 5–10 times more abundant in the hippocampus than in the cortex. In Figure 2C, 2D are displayed representative pictures of Congo-Red stained brain sections from TgAPP and TgAPP/PLTP–/– mice, with arrows pointing to amyloid plaques.

### Nesting behavior and spatial reference memory assessment in TgAPP mice with or without PLTP deficiency

**Figure 3:**
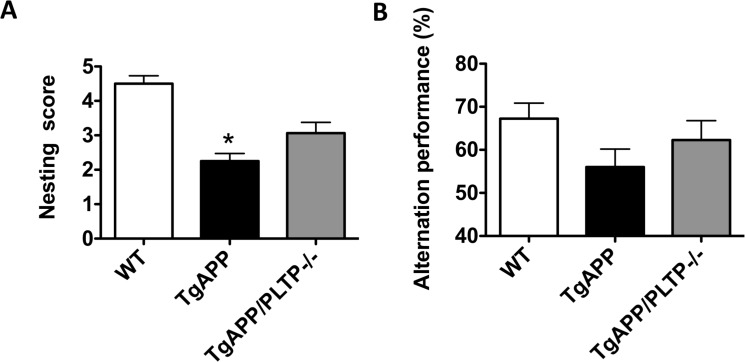
Behavioral analysis of TgAPP and TgAPP/PLTP–/– mice (**A**) Nest construction was quantified in 6 months old WT (*n* = 6), TgAPP (*n* = 6) and TgAPP/PLTP–/– (*n* = 5) female mice. Cotton nestlets (Plexx^®^) were provided as nesting material (1/cage). The nests were scored by two independent observers blind to the group identity, according to the following scale: 0 = undisturbed; 1 = disturbed; 2 = flat nest; 3 = cup-shaped nest; 4 = incomplete dome; 5 = complete dome. ^*^p < 0.05 *vs* WT (Kruskal–Wallis test with Dunns *post-hoc* analysis). (**B**) Short-term spatial reference memory was assessed in 6 months old WT (*n* = 11), TgAPP (*n* = 10) and TgAPP/PLTP–/– (*n* = 5) female mice through the measurement of spontaneous alternation percentage in the Y maze. One-way ANOVA : F_(2,23)_= 2.25, *p* > 0.05. Results are expressed as mean ± SEM.

Nest construction is an affiliative, social behavior that was reported to be impaired in various mouse models of AD [25–27]. To evaluate nest quality we used a 5-point rating scale. The average score of the nest quality of WT, TgAPP and TgAPP/PLTP–/– mice is shown in Figure 3A. Nest construction ability was significantly impaired in TgAPP mice compared to WT mice (*p* < 0.05), and a partial restoration of this capacity was observed in TgAPP/PLTP–/– mice.

We next assessed spatial reference short-term memory in WT, TgAPP and TgAPP/PLTP–/– mice in the Y maze paradigm. As shown in Figure 3B, the spontaneous alternation performances of TgAPP mice were non-significantly reduced compared to those of WT mice, and a tendency towards an increase was observed in TgAPP/PLTP–/– mice. The exploratory activity, assessed through the total number of arm entries, was not significantly different between WT, TgAPP and TgAPP/PLTP–/– animals (data not shown).

### PLTP deletion modulates neprilysin (NEP) but not Insulin-degrading-enzyme (IDE) expression in brain tissue

**Figure 4:**
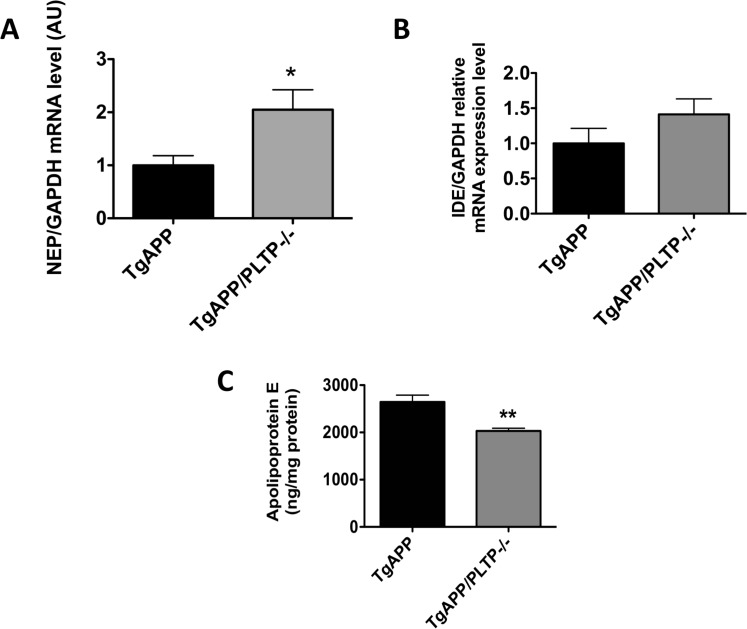
Protein-dependent amyloid beta clearance in TgAPP and TgAPP/PLTP–/– mouse brains The expression of the amyloid-beta degrading enzymes NEP (**A**) and IDE (**B**) was determined by semi-quantitative real-time PCR in purified mRNA preparations obtained from hemibrain homogenates of 6 months old TgAPP (*n* = 8) and TgAPP/PLTP–/– (*n* = 7) mice. Results were normalized using the expression level of GAPDH. Apolipoprotein E levels (**C**) were measured in hemibrain homogenates of TgAPP (*n* = 8) and TgAPP/PLTP–/– (*n* = 7) mice using an ELISA kit and were normalized to protein concentrations. Results are expressed as mean ± SEM. ^*^p < 0.05, ^**^p < 0.01 *vs* TgAPP mice (Student’s *t* test).

To address whether PLTP deletion can modulate Aβ clearance mechanisms, we first measured the mRNA expression of the main Aβ-degrading enzymes, NEP and IDE in brain extracts by real-time PCR. As shown in Figure 4A and 4B), PLTP deletion is associated with a significant, 2-fold increase in NEP expression in brain homogenates (*p* < 0.05). The expression of IDE is mildly but not significantly increased in TgAPP/PLTP–/– compared to TgAPP mouse brains.

### Brain apolipoprotein E levels are reduced in TgAPP/PLTP–/– compared to TgAPP mice

Apolipoprotein E, the most abundant apolipoprotein expressed in the CNS and the primary genetic risk factor for the vast majority of AD cases in humans [28], was assayed by ELISA in hemibrain homogenates. As shown in Figure 4C, a significant, 23% decrease in apolipoprotein E levels was measured in TgAPP/PLTP–/– brains compared to their TgAPP littermates (*p* < 0.01).

### PLTP deletion reduces astrogliosis and IL-6 levels in TgAPP mice

**Figure 5:**
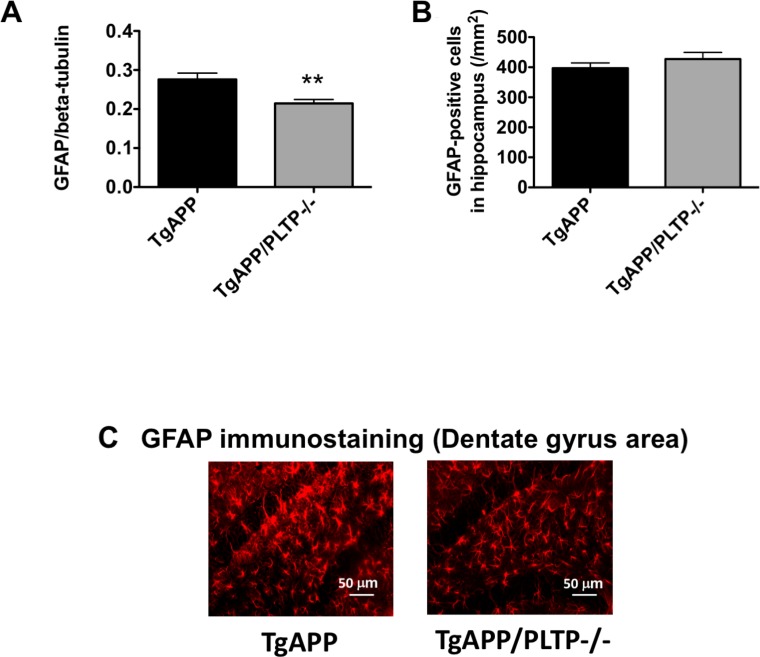
Quantification of astrogliosis in TgAPP and TgAPP/PLTP–/– brain tissues (**A**) The expression of the astrocyte activation marker GFAP was quantified in brain homogenates from 6 months old TgAPP (*n* = 7) and TgAPP/PLTP–/– mice (*n* = 6) by Western Blot. (**B**) Tissue sections from TgAPP (*n* = 5) and TgAPP/PLTP–/– mice (*n* = 5) were stained with anti-GFAP antibodies to detect astrogliosis and observed by fluorescence microscopy. For each animal, GFAP-positive cells were counted in 3 fields (1 in the dentate gyrus, 1 in CA1, 1 in CA3) from 4 different sections/animal. ^**^p < 0.02 *vs* TgAPP mice (Student’s *t* test). (**C**) Representative images of the staining observed in each group.

To determine whether astrogliosis was affected by deletion of the PLTP gene, GFAP was quantified by Western Blot in hemibrain homogenates and GFAP-positive cell numbers were determined in brain sections from TgAPP and TgAPP/PLTP–/– mice by immunohistochemistry. As shown in Figure 5A, 5B), a significant, 22% decrease in GFAP signal intensity was measured in brain homogenates from TgAPP/PLTP–/– compared to TgAPP mice (*p* < 0.02), while astrocyte numbers were similar in both groups. A representative picture of GFAP immunostaining in the dentate gyrus area of TgAPP and TgAPP/PLTP–/– mice is presented in Figure 5C. The observed decrease in astrogliosis is suggestive of a reduced neuroinflammatory response in TgAPP/PLTP–/– mice.

To further address the impact of PLTP deficiency on brain inflammatory state, the prototypic pro-inflammatory cytokines interleukin 6 (IL-6), interleukin 1-beta (IL-1beta), and tumor necrosis factor alpha (TNF-alpha) were assayed in brain homogenates fromTgAPP and TgAPP/PLTP–/– mice. As shown in Table 1, a significant, more than two-fold decrease in IL-6 levels was measured in TgAPP/PLTP–/– compared to TgAPP mouse brains (0.43 ± 0.07 *vs* 0.99 ± 0.08 pg/mg protein, respectively, *p* < 0.001), while no significant difference was measured for TNF-alpha and IL-1beta.

**Table 1:** Cytokine levels in TgAPP (*n* = 5) and TgAPP/PLTP–/– (*n* = 7) mouse brains

	TgAPP	TgAPP/PLTP–/–
IL 6 (pg/mg protein)	0.99 ± 0.08	0.43 ± 0.07^**^
IL 1beta (pg/mg protein)	1.14 ± 0.28	1.11 ± 0.29
TNF alpha (pg/mg protein)	0.1 ± 0.01	0.11 ± 0.01

Results correspond to mean ± SEM. (^**^p < 0.001 vs TgAPP, Mann Whitney test).

### PLTP deletion modifies microglial activation state and phagocytic activity in the brain of TgAPP mice

**Figure 6:**
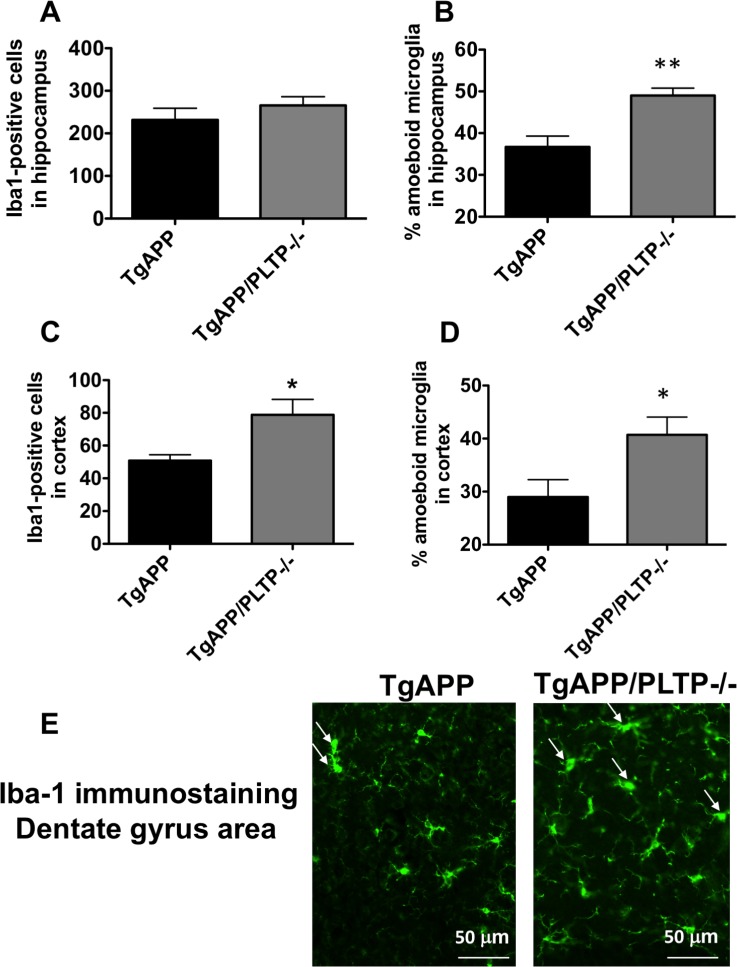
Quantification and characterization of microglial cells in the brain tissue of TgAPP and TgAPP/PLTP–/– mice Tissue sections from 6 months old TgAPP (*n* = 5) and TgAPP/PLTP–/– mice (*n* = 5) were stained with anti-Iba-1 antibodies to detect microglial cells, and observed by fluorescence microscopy. For each animal, Iba-1-positive cells were counted in 3 fields of the hippocampus (1 in the dentate gyrus, 1 in CA1, 1 in CA3) (**A**) and in 1 field of the parietal cortex (**C**) from 4 different sections. Two distinctive microglial phenoytpes were manually discriminated in the hippocampus (**B**) and parietal cortex (**D**) of TgAPP and TgAPP/PLTP–/– mice as described in the ‘Materials and Methods’ section, and the percentage of activated microglial cells was calculated. A representative Iba-1 staining in the dentate gyrus area of TgAPP and TgAPP/PLTP-/- brains is shown in (**E**), with arrows pointing to the activated microglial cells. Results are expressed as mean ± SEM. ^*^p < 0.05, ^**^p < 0.005 *vs* TgAPP mice (Student’s *t* test).

Microglia are considered as the predominant immune cells of the brain. They are tasked with the phagocytosis of CNS material including amyloid plaques, and are known to play a major role in AD pathogenesis [29, 30]. We sought to determine the effect of PLTP deletion on the activation state and functional phenotype of microglial cells. We quantified microglial cells number and characterized their morphology within the hippocampus and the parietal cortex of TgAPP mice and TgAPP/PLTP–/– mice (Figure 6). Our immunohistochemical analysis revealed a significant increase in the number of microglial cells (Iba1-positive) in the temporal cortex (*p* < 0.05), but not in the hippocampus of TgAPP/PLTP–/– mice compared to TgAPP mice (Figure 6A and 6C). Activated microglia are typified by an enlarged soma, short processes, and decreased branching [31]. We quantified the relative amounts of activated versus resting microglial cells in the hippocampus and parietal cortex of TgAPP and TgAPP/PLTP–/– mice. As shown in Figure 6B and 6D), the percentage of activated microglia was significantly increased in the hippocampus (*p* < 0.005) as well as in the the temporal cortex (*p* < 0.05) of TgAPP/PLTP–/– mice compared to TgAPP mice. Figure 6E is a representative picture of Iba-1 immunostaining in the dentate gyrus area of TgAPP and TgAPP/PLTP–/– mouse brains, illustrating the increased proportion of activated microglial cells in the context of PLTP deficiency.

**Figure 7:**
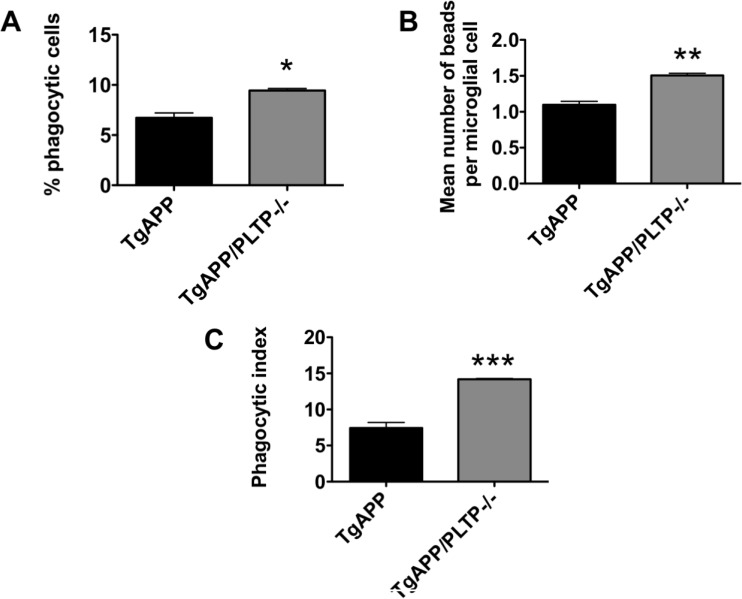
Phagocytosis ability of microglial cells from TgAPP and TgAPP/PLTP–/– mice Phagocytosis experiments were conducted with acutely isolated brain slices. Slices were incubated with FBS-coated fluorescent latex microspheres for 60 min and phagocytic activity of microglial cells was determined after Iba-1 immunostaining. The percentage of Iba-1 positive phagocytic cells (**A**), the mean number of beads per microglial cell (**B**), and the phagocytic index (**C**) were determined in brains slices from TgAPP (*n* = 3) and TgAPP/PLTP–/– (*n* = 3) mice. Results are expressed as mean ± SEM. ^*^p < 0.01, ^**^p < 0.005, ^***^p < 0.001 *vs* TgAPP mice (Student’s *t* test).

To determine whether PLTP is involved in microglial phagocytosis, we used acute brain slices and determined phagocytosis using FBS-coated fluorescent latex beads. Microglial cells were identified by Iba-1 immunostaining. Three independent experiments were performed; in each experiment one TgAPP brain and one TgAPP/PLTP–/– brain were sliced, and results were analyzed by a paired Student's *t* test. As shown in Figure 7A and 7B), both the percentage of phagocytic cells (+40%, *p* < 0.01) and the mean number of phagocytosed beads per microglial cell (+37%, *p* < 0.005) were increased in brain slices from TgAPP/PLTP–/– compared to TgAPP mice, resulting in an almost 2-fold increase (+91%) of the phagocytic index (*p* < 0.001) (Figure 7C).

## DISCUSSION

The incidence of Alzheimer's disease is expected to double in the next 20 years and will contribute to heavy social and economic burden. Currently, no cure exists and with the recent failure of clinical trials of drugs targeting solely Aβ, there is an urgent need to define new targets and develop alternative therapeutic strategies. In the present study, we provide evidence that cerebral Aβ peptide deposition is markedly reduced when PLTP is deleted in a hAPP transgenic mouse model of AD, which can be explained by an increase in Aβ clearance mechanisms.

There is growing evidence supporting the hypothesis that AD starts to develop due to an imbalance between Aβ production and removal. Whereas familial early-onset AD appears primarily related to overproduction of Aβ, sporadic late-onset AD may result from a dysfunction in the clearance system and/or the formation of hard-to-clear Aβ aggregates [32–35]. In the present study, we observed no difference in the expression of the human APP transgene, and a small, non significant increase in Aβ_1–42_ peptide levels were measured in soluble fractions of brain homogenates from TgAPP/PLTP–/– compared to TgAPP animals. These observations indicate that reduced expression or amyloidogenic processing of APP are unlikely to account for the decreased amount of Congo Red-positive plaques in this transgenic setting. Tong *et al.* reported in another AD model that PLTP deficiency is associated with a disrupted APP turnover and an enhancement of the amyloidogenic pathway, leading to increased soluble Aβ peptides and intracellular accumulation of Aβ [36]. Although the reason of the discrepancy between these observations and our findings is not clear at this time, it is worthy of note that their study was conducted in three month-old animals, which corresponds to a very early stage of the amyloid pathology where amyloid deposition has not appeared yet; moreover, as reported earlier for the membrane receptor TREM2 [37], there could be a stage-dependent differential effect of PLTP on the pathology. Another hypothesis is that the discrepancy relies on differences in the promoter, expression and metabolism of the transgene product, human APP protein, between the J20 (PDGF promoter) and APP/PS1 (Thy1 promoter) models. Interestingly, a differential effect of the genetic ablation of ABCA7, a transmembrane lipid carrier, was also observed in the APP/PS1 and J20 models. Thus, in 2013, Kim *et al*. reported that ABCA7 deficiency in J20 mice is associated with a reduced phagocytic capacity of microglial cells, affecting Aβ clearance and leading to increased deposition. They did not observe any alteration in APP processing in this model. One year later, Sakae *et al.* reported that increased amyloidosis in APP/PS1 mice with ABCA7 deficiency is associated with increased APP processing and Aβ formation, with no impact on its clearance [38]. Thus, one can hypothesize that due to intrinsic differences between the J20 and APP/PS1 models, the impact of a genetic modification on APP processing is not the same. It is worthy of note that APP processing is markedly accelerated in APP/PS1 mice due to the presence of the PS1 transgene, which may interfere with the action of the endogenous cleaving enzymes and their modulators. Finally, the differences could also be explained by environmental factors in animal facilities modifying the immunological status of the animals.

More generally, it is important to keep in mind that results obtained using AD models should always be interpreted with caution, since each model, depending on how it was designed, provides a unique pathophysiological context, with its own specificities and limitations. Most importantly, species differences between rodents and humans in terms of neuroanatomy, genetics, behaviors, and immune functions force to limit extrapolations.

Several clearance mechanisms tightly regulate Aβ levels in the brain. One of them relies on the action of specialized cells of the CNS called microglia. Emerging evidence suggests that activated microglial cells can work as a double-edged sword with regards to neurodegeneration. Thus, they can either produce and secrete pro-inflammatory mediators (including IL-1beta, TNF-alpha, and IL-6), or instead release protective factors (triggering anti-inflammatory and immunosuppressive responses) and phagocytose Aβ aggregates [39, 40]. The important role of microglia in the phagocytic clearance of Aβ aggregates in AD mouse models and in Aβ42-immunized human AD patients has been clearly demonstrated (for review, see [41]). Recent human genome-wide association studies (GWAS) and experimental studies have identified that functional microglia impairment would play a dominant role in the accumulation of amyloid plaques and the pathophysiology of AD [42].

Although PLTP was mostly studied for its role in lipid metabolism and cardiovascular disease, recent evidence suggests that it also plays a major role in inflammation and modulation of the immune response in the periphery. PLTP-deficient mice were shown to display an anti-inflammatory phenotype (e.g. lower circulating levels of IL-6) [43, 44], and reduced expression level of IL-6 and infiltrating macrophages in aortic tissue of PLTP–/– mice as compared to wild type mice was recently observed in an experimental model of abdominal aortic aneurysm [45]. In macrophages, PLTP exerts a direct anti-inflammatory role through direct interaction with the ATP-binding cassette transporter A1 (ABCA1) and subsequent activation of the JAK2/STAT3 pathway [14]. PLTP also acts as an essential acute phase protein to suppress LPS-induced inflammation *in vivo* and *in vitro* via its LPS neutralization capacity [10, 12], and PLTP–/– mice show increased mortality after LPS injection compared to wild-type mice [11]. In addition, reduced PLTP expression or activity were shown to enhance the inflammatory response in cigarette smoke extract-exposed lung and small airway epithelial cells [46]. PLTP also modulates the acquired immune response, since our group recently demonstrated that PLTP deficiency is associated with a shift of T*helper* (Th) lymphocytes polarization towards the anti-inflammatory subset, Th2 [13].

In the present study, we provide the first evidence of an involvement of PLTP in the modulation of innate immune reponses in the CNS. We showed that PLTP deletion is associated with a shift in microglial cells phenotype towards the ameboid form, with a marked increase in their phagocytic capacity. Since a sharp reduction of amyloid deposits was observed in TgAPP/PLTP–/– compared to TgAPP mice, our finding is in line with the current idea that switching microglia phenotype plays a dominant role in limiting the expansion of amyloid plaques [47]. The reason why PLTP modifies the phenotype of microglial cells is not clear at this time and will deserve further investigation. Our group previously demonstrated that peritoneal macrophages from PLTP-deficient mice have an increased capacity to phagocytose oxidatively modified low-density lipoproteins and become foam cells, a property related to increased intracellular oxidative stress [9]. Since cerebral oxidative stress is significantly higher in PLTP-deficient mice than in WT mice [5], a similar mechanism may account for the increased ability of microglial cells to phagocytose Aβ aggregates observed in the AD context. Interestingly, we measured a marked reduction of IL-6 level in brain homogenates from TgAPP/PLTP–/– compared to TgAPP mice, which is concordant with earlier observations made in the periphery [43, 44]. IL-6 promotes microglial pro-inflammatory activities [48] and induces the synthesis of acute phase proteins [49]. Several studies have reported increased serum and plasma levels of IL-6 in patients with AD as compared to controls [50, 51]. Whether reduced IL-6 level is an endophenotype of PLTP-deficient mice and contributes to the increased phagocytic potential of microglial cells, or whether it is a consequence of altered microglial polarization will warrant further investigation.

Beyond microglial cells, several enzymes also participate to the degradation of Aβ deposits in the brain. Among those enzymes are several zinc-proteases: neprilysin (NEP) and the Insulin-Degrading Enzyme (IDE). During the ageing process, and under certain pathological conditions (e.g. ischemia and stroke), the expression and activity of these enzymes decline, which leads to a deficit of Aβ clearance and its accumulation in the brain. In agreement with this notion, pharmacological inactivation [52] or genetic ablation [53, 54] of NEP in hAPP mice increased total Aβ levels in the brain and plasma, whole brain and hippocampal levels of Aβ dimers, Aβ deposition, plaque-related pathology, deficits in synaptic plasticity, and impairments in hippocampus-dependent memory. In contrast, viral vector-mediated [55, 56] or transgene-mediated [57, 58] overexpression of NEP reduced cortical Aβ levels, Aβ deposition, and plaque-related pathology. In the present study, we observed a significant increase in the mRNA expression of NEP in hAPPTg/PLTP–/– mice compared to hAPPTg mice, which may also contribute to the reduced amyloid deposition.

We measured a significant decrease in apolipoprotein E levels in brain homogenates from TgAPP/PLTP–/– compared to TgAPP mice. This finding corroborates previous *in vitro* findings showing that PLTP induces apolipoprotein E secretion in primary human astrocytes, and that CSF apoE levels correlate with CSF PLTP activity [23]. A direct interaction between apolipoprotein E and PLTP, leading to PLTP activation, has also been demonstrated, suggesting a strong functional link between the two proteins [59]. Apolipoprotein E is the primary genetic risk factor for the vast majority of sporadic, late-onset AD cases in the population. Although several mechanisms for the effect of the apoE4 variant on AD pathogenesis have been proposed, the primary pathway appears to be the differential effects of apoE isoforms on Aβ aggregation and clearance [60, 61]. In addition to isoform status, it appears that the amount of apoE also plays a role in determining the extent of Aβ accumulation in the brain. There has been intense debate as to whether potential AD therapeutics should increase or decrease apoE levels. Based on the strong association between ApoE and Aβ in the brain, ApoE was suggested as an Aβ-binding protein that induces a pathological β sheet conformational change in Aβ [62]. Initial *in vivo* studies clearly indicated that the deletion of the endogenous murine *Apoe* gene causes a dramatic decrease in fibrillar and total Aβ deposition in APP transgenic mouse models [63–65]. Recently, ApoE was shown to interfere with the uptake of amyloid β oligomers by astrocytes and fibrillar amyloid β by microglial cells [66]. Thus, reduced apolipoprotein E concentration in the brain tissue of TgAPP/PLTP–/– mice is another feature that may contribute to the reduced accumulation of amyloid β aggregates.

The finding that PLTP deletion did not counteract memory deficits in the J20 mice despite a strong reduction of amyloid deposition might be due to the fact that soluble Aβ levels were not significantly changed. It is noteworthy however that although the findings did not reach statistical significance, PLTP deficiency tends to improve nest building activity, an indicator of well-being and executive functioning. It has been reported that memory loss and insoluble Aβ aggregates were not connected in APP transgenic mice (Tg2576) and transgenic mice co-expressing mutant presenilin-1 and APP, respectively, which suggested that earlier Aβ aggregates disrupted cognition [67–69]. In 2008, long-term clinical follow-up, post-mortem neuropathological examination of Aβ_1–42_ immunization in AD patients showed that although immunization with Aβ_1–42_ resulted in reduction of amyloid plaques in patients with AD, this reduction did not prevent progressive neurodegeneration and cognitive decline [70]. Thus, the notion that plaque loads are unreliable indicators of AD-related functional impairments is now widely accepted. In the J20 model, memory impairment appears at the age of 2 months, well before amyloid deposition [24], and is not dependent of disease progression, which suggests that in these mice like in other AD models, soluble multimeric forms of amyloid β rather than amyloid deposits might initiate the synaptic and neuronal dysfunction leading to cognitive decline [68].

In summary, we have shown that PLTP deletion is associated with a significant reduction of Aβ deposits and astrogliosis in the brain. This phenotype can be explained at least in part by a rise of Aβ clearance through an increase in the microglial phagocytic activity and the expression of NEP. Our findings suggest that PLTP acts as a negative determinant of plaque clearance and that over the lifespan, elevated PLTP activity could lead to a higher Aβ load in the brain. In line with the present study, it would be worthwhile to determine the impact of PLTP overexpression in AD mice on amyloid deposition and cognitive functions.

## MATERIALS AND METHODS

### Animals

Hemizygous hAPP_SwInd_ transgenic (J20) mice [24] were purchased from the Jackson Laboratories, and *PLTP^−/−^* mice were generated as described by Jiang *et al*. [71]. A Material Transfer Agreement (#UM140255-01) was signed with the Gladstone Institute for the use of the J20 mice. All mice were on the C57BL/6J background and backcrossed >10 generations. The genotyping of the J20 and *PLTP^−/−^* mice have been described previously [24, 71]. J20 mice and *PLTP*^−/−^ mice were crossed to generate heterozygous TgAPP*/PLTP^+/−^* breeders. These mice were mated to obtain TgAPP*/PLTP^+/+^* (referred to as TgAPP) and TgAPP/*PLTP^−/−^* (referred to as TgAPP/PLTP–/–) mice. Six months old animals were used throughout this study. Wild-type (WT) mice were used as controls for behavioral testing. All mice had free access to water and food, and they were fed a standard chow diet (A03, SAFE Diets, France). This project follows the specific French national guidelines on animal experimentation and well-being, and ethics approval was acquired from the Ministère de l’Education Nationale, de l’Enseignement Supérieur et de la Recherche, under the reference 00965.03.

### Tissue preparation for histological and biochemical analyses

Mice were anesthesized with a ketamine/xylazine mixture and euthanized by cardiac perfusion with PBS. The brains were carefully removed and sagittally divided; the left hemisphere was snap frozen and stored at –80°C; the right hemisphere was fixed in 4% paraformaldehyde for 96 hours at 4° C, then transferred to a 30% sucrose solution for 7 days. Tissues were then included in a block of OCT compound (Tissue-Tek^®^, Sakura Finetek, USA) and quickly frozen in acetone chilled on dry ice. Frozen blocks were mounted on a cryostat (Leica, France) and 25 μm-thick coronal sections were collected and stored at –20° C in anti-freeze solution. Frozen, left hemi-brains were homogenized using an ultrasound probe in phosphate buffer containing protease inhibitors (Complete Ultra, Roche, Switzerland). Homogenates were centrifuged at 20,000 × g for 30 min at 4° C and the supernatant (soluble fraction) was stored at –80° C until use. The pellet was resuspended in 0.5 ml of a 70% formic acid solution, sonicated, and stirred for 2 h at 4° C. The solution was then neutralized with 6.5 ml of a 1.5 M Tris-HCl solution at pH 11. This solution was used as the insoluble protein fraction and stored at –80° C until use. Protein concentrations were measured using the bicinchoninic acid method (Pierce BCA kit/ Thermofisher Scientific, France).

### Quantification of amyloid plaques

Free-floating coronal sections were rinsed with PBS to remove cryoprotectants. The sections were then stained for amyloid plaques using the Amyloid Stain/Congo Red kit (Sigma-Aldrich, France), mounted on glass slides, and counterstained with hematoxylin/eosin. Congo red-stained sections were visualized and photographed using a DM2500 microscope fitted with a DFC495 high-resolution camera (Leica Microsystem, France) and the LAS Core image analysis software (Leica Microsystem, France). Digitized images were acquired using a 40X objective, transformed into TIFF files and brought to the same level of contrast and sharpness using the software. At least 12 sections were studied from each brain, taken from the anterior hippocampus level, with intervals of 100 μm. The investigator was blinded to the identity of the mouse brains.

### Amyloid-beta peptides quantification

Human amyloid-β_1–40_ and amyloid-β_1–42_ peptides, as well as aggregated species were quantitated in the soluble fraction of brain homogenates using ELISA kits (Cloud-Clone Corp., USA) and following the manufacturer's recommendations. Results were normalized to protein concentrations.

### Apolipoprotein E assay

The apolipoprotein E content was quantified in brain homogenates using an ELISA kit (Cloud Clone Corp.) and following the manufacturer's instructions. Apolipoprotein E levels were normalized to protein concentrations.

### Western blotting

After adjustment to the same protein concentration (5 μg/ml), samples were diluted (1:1) in Laemmli buffer, boiled for 5 min and loaded on a 10% polyacrylamide gel. After protein transfer, the membrane was blocked in 5% non-fat dry milk and was incubated in primary antibody overnight at 4°C, then incubated for 2 h at room temperature with IRDye secondary antibodies (LI-COR Biosciences, Germany). Signal intensities were quantified using an Odyssey Fc quantitative fluorescence imaging system (LI-COR Biosciences).

Primary antibodies used were the following : anti β-amyloid 1–16 monoclonal antibodies (6E10 clone, 1:1000; BioLegend/Ozyme, France) to detect full-length human APP, anti-GFAP antibodies (1/1000, Sigma-Aldrich, France) and rabbit anti-BACE1 antibodies (1:1000; Cell Signalling Technologies/Ozyme, France). Anti-βtubulin (β-tub) antibodies (1:10000; Sigma-Aldrich) were used for normalization.

### RNA extraction and real-time PCR

Total RNA was isolated from brain homogenates using Nucleospin RNA extraction kit (Macherey Nagel, Germany) according to the manufacturer's instructions. Neprilysin (NEP) and insulin-degrading enzyme (IDE) mRNA levels were quantified by reverse transcription followed by real-time PCR using a Light Cycler 480 II detection system (Roche, Switzerland), at the GenomiX qPCR facility of the Montpellier University. Amplification was undertaken using the Light Cycler SYBR Green I Mix (Roche) following the instructions provided by the manufacturer. GAPDH was used as a reference gene and results were expressed as the ratio between the mRNA expression level of target and reference genes.

Oligonucleotide primers used for cDNA amplification were as follows:

**Table:** 

Gene	Sense	Antisense
GAPDH	TGCCATTTG CAGTGGCAA	TTCCAGAGG GGC CATCCA
NEP	GAGCCCC TTACTAGG CCTGTGT	CTCGATTC AGACATAG GCTTTCTAAA
IDE	CCGGCCAT CCAGAGA ATAGAA	ACGGTATT CCCGTTTG TCTTCA

### Cytokine assays

Interleukin 6 (IL-6), interleukin 1beta (IL-1beta), and Tumor Necrosis Factor alpha (TNF alpha) were assayed in brain homogenates prepared in 20 mM Tris, 150 mM NaCl, 10 mM EDTA (pH 7.5) buffer containing protease inhibitors and 0.1% Triton-X100. Assays were performed using ELISA Ready-SET-Go kits from eBiosciences (USA), and data were normalized to total protein concentrations.

### Immunohistochemistry and microglial activation state classification

Analysis of the astrocytic activation marker GFAP and of the microglial marker Iba-1 was conducted by fluorescence immunohistochemistry. Sections were rinsed in PBS, blocked 1h at room temperature with 3% goat serum, and incubated overnight at 4°C with a mouse anti-GFAP antibody (1/1000, Sigma-Aldrich, France) and a rabbit anti-Iba-1 antibody (1/750, Wako Chemicals, Japan). Sections were then incubated for 2 h with goat anti-mouse fluorescent (Cy3) (Jackson Immunoresearch, USA) and goat anti-rabbit fluorescent (AlexaFluor 488) (Thermofisher Scientific, France) secondary antibodies. Nuclei were counterstained with 4′,6′-diamino-2-phenylindole (DAPI) (Molecular Probes/Thermofisher Scientific, France). The immunostaining specificity was determined with the same protocol but by incubating control sections with the secondary antibody alone.

Four sections per animal were used for the cell countings. Three fields per section (1 in dentate gyrus, 1 in CA1, 1 in CA3) were used to count GFAP-positive and Iba-1 positive cells and to assess the morphology of microglial cells in the hippocampus. Iba-1 positive cells were counted in one field of the temporal cortex.

Two distinctive microglial phenotypes were manually discriminated in the brain of TgAPP and TgAPP/PLTP–/– mice using the following criteria : Type 1 : ramified cells with small soma and long processes (quiescent cells); Type 2 : ameboid cells with enlarged soma, short processes, and decreased branching (activated cells) [31, 72]. Microglial cells classification was performed by an observer blind to the group identities.

### *In situ* phagocytosis assay with opsonized latex microspheres

An *in situ* phagocytosis assay in adult cortical slices was performed as previously described [73]. Briefly, freshly isolated brains from 6–9 months old TgAPP and TgAPP/PLTP–/– mice were cut into 150-mm coronal slices with a vibratome (Microm HM650V). Slices were placed onto a nylon grid and incubated in ice-cold Krebs buffer (124 mmol/L sodium chloride, 3.5 mmol/L potassium chloride, 25 mmol/L sodium bicarbonate, 1.25 mmol/L sodium dihydrogen phosphate, 1 mmol/L calcium dichloride, 2 mmol/L magnesium sulfate, 10 mmol/L glucose) bubbled with carbogene (95% CO2, 5% O2) for 2 h. Red fluorescent carboxylated microspheres (2.0 mm diameter, Sigma-Aldrich) were coated with fetal calf serum, and 500 ml Krebs buffer containing 8.10^6^ beads was applied on each brain slice and incubated 60 min at 37°C in an incubator. After incubation, slices were washed three times with ice-cold PBS, fixed with an AntigenFix solution (Diapath, Italy) and immunostained for microglia with anti-Iba1 antibodies as described above.

Confocal laser scanning microscopy was performed on a Leica SPE confocal microscope with the LAS AF software, on the MRI (Montpellier Ressources Imagerie) facility of the Montpellier University. Z-stacks of 10 mm-thickness were performed in acute brain slices using a 40×/1.15 objective with a step size of 1 mm beginning from the top of the slice, where the microspheres are located. Between 4 and 6 slices were analyzed per mouse brain. Total number of microglial cells, number of phagocytic microglia, and total number of phagocytosed beads per microglial cell were counted using the Image J software (W. Rasband, NIH, USA). The data are presented as percentage of phagocytic microglial cells, and mean number of beads per microglial cell. The resulting phagocytic index was calculated as the product of the 2 parameters.

### Nesting test

Female mice were placed individually in polysulfone cages with bedding about 1 h before the dark phase. Cotton nestlets (Plexx^®,^ The Netherlands) were provided as nesting material (1/cage), and nesting behavior was assessed the next morning. The nests were scored by two independent observers blind to the group identity, according to the following scale: 0 = undisturbed; 1 = disturbed; 2 = flat nest; 3 = cup-shaped nest; 4 = incomplete dome; 5 = complete dome [74].

### Spatial reference memory assessment

Only female mice were used for behavioral experiments, which were performed between 10:00 am and 05:00 pm. Spontaneous alternation behavior, a measure of short-term spatial reference memory, was tested using a Y-maze following a previously described protocol [7]. The percentage of alternation was calculated as (actual alternations/maximum alternations) ×100.

### Statistical analysis

Data are presented as mean ± SEM. Spontaneous alternation performances were analyzed using a one-way ANOVA (*F* value). Nesting scores were analyzed using a non parametric Kruskal-Wallis test, with Dunns *post-hoc* analysis. All other data were analyzed using the Student's *t* test (GraphPad Prism 5.0). Normality of the distributions was assessed using the Kolmogorov-Smirnov test. *P* < 0.05 was considered significant.

## Abbreviations

GFAP: Glial Fibrillary Acidic Protein
PLTP: Phospholipid Transfer Protein
CNS: Central Nervous System
CSF: Cerebrospinal Fluid
Aβ: Amyloid beta
AD: Alzheimer's Disease
APP: Amyloid Precursor protein
NEP: Neprilysin
IDE: Insulin-Degrading Enzyme
WT: Wild-type
TNF-alpha: Tumour Necrosis Factor-alpha
IL-6: Interleukin 6
IL-1beta: Interleukin 1-beta
LPS: lipopolysaccharides.
